# Homeostatic regulation of extracellular signal-regulated kinase 1/2 activity and axonal K_v_7.3 expression by prolonged blockade of hippocampal neuronal activity

**DOI:** 10.3389/fncel.2022.838419

**Published:** 2022-07-28

**Authors:** Brian C. Baculis, Harish Kesavan, Amanda C. Weiss, Edward H. Kim, Gregory C. Tracy, Wenhao Ouyang, Nien-Pei Tsai, Hee Jung Chung

**Affiliations:** ^1^Neuroscience Program, University of Illinois at Urbana-Champaign, Champaign, IL, United States; ^2^Department of Molecular and Integrative Physiology, University of Illinois at Urbana-Champaign, Champaign, IL, United States; ^3^Beckman Institute for Advanced Science and Technology, University of Illinois at Urbana-Champaign, Champaign, IL, United States; ^4^Institute of Genomic Biology, University of Illinois at Urbana-Champaign, Champaign, IL, United States

**Keywords:** homeostatic plasticity, ankyrin-G, ERK, K_v_7, axon initial segment

## Abstract

Homeostatic plasticity encompasses the mechanisms by which neurons stabilize their synaptic strength and excitability in response to prolonged and destabilizing changes in their network activity. Prolonged activity blockade leads to homeostatic scaling of action potential (AP) firing rate in hippocampal neurons in part by decreased activity of N-Methyl-D-Aspartate receptors and subsequent transcriptional down-regulation of potassium channel genes including *KCNQ3* which encodes K_v_7.3. Neuronal K_v_7 channels are mostly heterotetramers of K_v_7.2 and K_v_7.3 subunits and are highly enriched at the axon initial segment (AIS) where their current potently inhibits repetitive and burst firing of APs. However, whether a decrease in K_v_7.3 expression occurs at the AIS during homeostatic scaling of intrinsic excitability and what signaling pathway reduces *KCNQ3* transcript upon prolonged activity blockade remain unknown. Here, we report that prolonged activity blockade in cultured hippocampal neurons reduces the activity of extracellular signal-regulated kinase 1/2 (ERK1/2) followed by a decrease in the activation of brain-derived neurotrophic factor (BDNF) receptor, Tropomyosin receptor kinase B (TrkB). Furthermore, both prolonged activity blockade and prolonged pharmacological inhibition of ERK1/2 decrease *KCNQ3* and *BDNF* transcripts as well as the density of K_v_7.3 and ankyrin-G at the AIS. Collectively, our findings suggest that a reduction in the ERK1/2 activity and subsequent transcriptional down-regulation may serve as a potential signaling pathway that links prolonged activity blockade to homeostatic control of BDNF-TrkB signaling and K_v_7.3 density at the AIS during homeostatic scaling of AP firing rate.

## Introduction

Activity-dependent changes in synaptic strength and intrinsic excitability occur rapidly or over a prolonged period in many different types of neurons in the brain, and could lead to unstable or saturated neural networks. Homeostatic plasticity counteracts these destabilizing changes by adjusting their synaptic strength and intrinsic excitability within a physiological range (Turrigiano, 2012). For example, prolonged activity blockade using voltage-gated sodium channel blocker, tetrodotoxin (TTX) leads to a homeostatic increase in action potential (AP) firing rate of hippocampal neurons in dissociated culture, slice culture, and *in vivo* (Aptowicz et al., 2004; Echegoyen et al., 2007; Lee et al., 2015). Homeostatic scaling of firing rate in cultured hippocampal neurons is associated with decreases in the transcripts of plasticity-related genes including brain-derived neurotrophic factor (*BDNF*) and the subunits of multiple potassium (K^+^) channels critical for regulating intrinsic excitability including *KCNQ3*/K_v_7.3 (Lee et al., 2015).

Neuronal K_v_7 channels are mainly composed of K_v_7.2 and K_v_7.3 (Delmas and Brown, 2005) which show overlapping expression in the hippocampus and cortex (Pan et al., 2006). They produce voltage-dependent slow activating and non-inactivating outward K^+^ current (*I*_*M*_) which potently suppresses repetitive and burst firing of APs (Brown and Passmore, 2009). They are inhibited by membrane depletion of phosphatidylinositol-4,5-bisphosphate (PIP_2_) upon activation of Gq/11-coupled receptors such as the M1 muscarinic acetylcholine receptor (Greene and Hoshi, 2017). K_v_7 channels also regulate resting membrane potential, AP threshold, and spike frequency adaptation, contribute to medium and slow afterhyperpolarization currents, suppress temporal summation, and mediate membrane potential resonance and intrinsic oscillations (Aiken et al., 1995; Yue and Yaari, 2004; Gu et al., 2005; Hu et al., 2007, 2009; Shah et al., 2008; Tzingounis and Nicoll, 2008). Subcellular, expression of K_v_7 channels is higher in the axonal plasma membrane compared to the somatodendritic surface of hippocampal neurons with the greatest enrichment at the axon initial segment (AIS) (Chung et al., 2006), which establishes neuronal polarity and serves as the site for AP initiation and modulation (Pan et al., 2006; Leterrier, 2018). The AIS localization of K_v_7 channels is mediated by ankyrin-G (Pan et al., 2006) and is critical for suppressing AP firing (Shah et al., 2008). However, whether a decrease in K_v_7 channel expression occurs at the AIS during homeostatic scaling of intrinsic excitability remains unknown.

Furthermore, the signaling pathway that mediates K_v_7 down-regulation during homeostatic scaling of intrinsic excitability is unclear. We have previously shown that homeostatic scaling of firing rate depends on a reduction in N-methyl-D-aspartate-type glutamate receptor (NMDAR) activity (Lee et al., 2015). Among multiple kinases activated downstream of NMDAR that mediate neuronal plasticity (Fan et al., 2005; Lin et al., 2008; Rosenkranz et al., 2009), one potential candidate is extracellular signal-regulated kinase 1/2 (ERK1/2) of the mitogen-activated protein kinase signaling pathway (Pearson et al., 2001). ERK1/2 signaling is required for visual cortical plasticity during monocular deprivation (Di Cristo et al., 2001), which is a model of homeostatic plasticity of excitatory synaptic strength (Ranson et al., 2012; Zhou et al., 2017). ERK1/2 also regulates the transcription of plasticity-related genes including *BDNF* (Adams and Sweatt, 2002; Thomas and Huganir, 2004), and BDNF is shown to prevent homeostatic scaling of intrinsic excitability by activating its receptor, Tropomyosin receptor kinase B (TrkB) (Desai et al., 1999).

In this study, we investigated if homeostatic scaling of intrinsic excitability involves the downregulation of K_v_7 channels at the AIS *via* a reduction in ERK1/2 signaling. We found that prolonged activity blockade in cultured hippocampal neurons transiently reduced ERK1/2 activity before decreasing TrkB activity, *BDNF* and *KCNQ3* transcripts, and the AIS expression of K_v_7.3 and ankyrin-G. Prolonged pharmacological inhibition of ERK1/2 was sufficient to induce the same reductions, suggesting ERK1/2 downregulation as a potential mechanism that mediates homeostatic control of *KCNQ3/*K_v_7.3 transcript and expression at the AIS.

## Materials and methods

### Materials

Chemical reagents used included Tetrodotoxin citrate (TTX, Tocris), 1,4-Diamino-2,3-dicyano-1,4-bis[2-aminophenylthio]butadiene (U0126, Tocris), and dimethyl sulfoxide (DMSO, Sigma). 10 mM TTX and 20 mM U0126 stock solutions were prepared using deionized water and DMSO, respectively. Antibodies used included anti-ERK1/2-pTyr^202/204^, anti-ERK1/2, anti-TrkA-pTyr^674/675^/TrkB-pTyr^706/707^, anti-TrkB, anti-GAPDH, anti-MAP2 (all from Cell Signaling), anti-Ankyrin-G (Neuromab), anti-K_v_7.3 (Alomone), horseradish peroxidase-conjugated secondary antibodies (Jackson ImmunoReserach Laboratory), and secondary antibodies conjugated to Alexa Fluor 488, 594, and 647 (Thermo Fisher Scientific).

### Experimental animals

All procedures involving animals were reviewed and approved by the Institutional Animal Care and Use Committee at the University of Illinois at Urbana-Champaign in accordance with the guidelines of the U.S. National Institute of Health (NIH).

### Hippocampal neuronal culture

Primary dissociated hippocampal neuronal cultures were prepared from Sprague–Dawley rat embryos at E18 as described (Brewer et al., 1993). Neurons were plated on Poly L-lysine (PLL) (0.1 mg/ml)-coated cell culture dishes for QPCR (2.3 × 10^6^ cells per 60 mm dish) and immunoblotting (0.6 × 10^6^ cells per 30 mm dish). For immunostaining, neurons were plated on PLL-coated 12 mm glass coverslips (1.0 × 10^5^ cells per coverslip) or 35 mm imaging dishes with a polymer coverslip bottom for high-end microscopy (5 × 10^5^ cells per dish, ibidi). Neurons at 10 days *in vitro* (DIV) were treated with TTX (1 μM) or U0126 (20 μM) for 24, 36, or 48 h (h) in culture media. The vehicle controls of TTX and U0126 were 0.1% H_2_O and 0.1% DMSO, respectively. For all drug treatments, drugs were refreshed after 24 h.

### qPCR

Total RNA was isolated using the RNeasy Kit (Qiagen). To synthesize cDNA, reverse transcription was performed using 2 μg of total RNA, random nanomers, dNTPs, M-MuLV reverse transcriptase, and RNase inhibitor. The resulting cDNA was subjected to qPCR using the StepOnePlus Real-Time PCR system (Applied Biosystems) with previously validated primers (Lee et al., 2015). Data were analyzed using the comparative threshold cycle (Ct) method (Schmittgen and Livak, 2008) and the internal control gene *GAPDH*. Following normalization to *GAPDH* cDNA levels, which is reflected in the ΔCt values, the relative mRNA quantification (RQ) of the fold change for each treatment compared to reference control was determined using the equation: RQ = 2^(–Δ Ct)^/2^(–Δ Ct reference)^.

### Immunoblot analysis

The hippocampal neuronal culture was lysed in ice-cold radioimmunoprecipitation assay buffer (50 mM Tris, 150 mM NaCl, 2 mM EDTA, 1% Triton X-100, 0.1% SDS, 0.5% deoxycholate) supplemented with Halt protease and phosphatase Inhibitor Cocktail (Thermo Fisher Scientific) as described (Lee et al., 2015). The resulting lysates were immunoblotted with primary antibodies for K_v_7.3 (1:500), ERK1/2-pTyr^202/204^, ERK1/2, TrkA-pTyr^674/675^/TrkB-pTyr^706/707^, TrkB, and GAPDH (all 1:1000). ImageJ software (NIH) was used to quantify the immunoblot band intensity and background. The background-subtracted band intensity of the protein of interest was first divided by that of the loading control GAPDH. The ratio (protein of interest/GAPDH) of the vehicle control was used as 100%, and the ratio of the drug treatment (TTX or U0216) was normalized to the vehicle control.

### Immunocytochemistry

Following drug treatment, neurons were washed with artificial cerebral spinal fluid (ACSF) containing drugs and fixed for 10 min with 4% paraformaldehyde (PFA) and 4% sucrose in PBS, whereas neurons in a 35 mm imaging dish (ibidi) were fixed for 3 min with 1% PFA and 4% sucrose in PBS. After blocking for 1 h in 10% normal donkey serum (NDS) and 0.2% Triton X-100 in PBS, neurons were incubated overnight with antibodies for ankyrin-G (1:500), K_v_7.3 (1:500), or MAP2 (1:500) in 3% NDS in PBS at 4°C. After neurons were washed in PBS and incubated for 2 h with secondary antibodies (1:200), they were thoroughly washed and mounted in Fluoro-Gel antifade mounting medium (Electron Microscopy Sciences).

### Imaging and image analysis

High-resolution 16-bit grayscale fluorescence images (1,920 × 1,216 pixels) were acquired using a Zeiss Axio Observer inverted microscope with a Zeiss AxioCam 702 mono Camera and ZEN Blue 2.6 software. To compare the fluorescence intensities of the neurons with different drug treatments, the images of each color were acquired using the same exposure time within one independent experiment. Image analysis was performed only on the healthy neurons using Fiji software (Schindelin et al., 2012). Neurons with regions containing fasciculation or overlapping axons were excluded.

The AIS is defined as an axonal initial segment that is immuno-positive to ankyrin-G. For image analysis, we focused on the ankyrin-G-positive segment of an axon that originates from the soma. The start of the AIS was identified as the point in the axon where the fluorescence intensity of ankyrin-G sharply increases by twofold within a few pixels. The end of the AIS was identified as the point in the axon where the ankyrin-G intensity sharply drops to the background axon intensity.

To obtain the raw integrated intensity value and length of the ankyrin-G-positive segment, we drew an ROI line (3 pixel thick) down the center of the axon from the start to the end of the ankyrin-G-positive segment under the “ankyrin-G” image channel. We then changed to the “K_v_7.3” image channel and obtained the raw integrated intensity of K_v_7.3 within the same ROI line. Background intensity was measured for each image channel as an area with no cells. Because the raw intensity values varied from one independent experiment to another (possibly due to the use of different lots of the purchased primary and secondary antibodies which might have affected labeling efficiency and/or fluorescence intensities), each background-subtracted intensity of ankyrin-G and K_v_7.3 from vehicle- and drug-treated neurons was normalized to its average intensity value of vehicle-treated neurons within each independent experiment as described (Zhang et al., 2020).

### Statistical analysis

Data are reported as mean ± SEM with individual data points. Statistical analysis was performed using OriginPro 2019. The Student’s two-tailed *t*-test was used to compare the two groups. One-way ANOVA and *post hoc* Tukey were used to compare groups ≥ 3. The priori value (*p*) < 0.05 was considered statistically significant.

## Results

### Prolonged activity blockade reduces extracellular signal-regulated kinase 1/2 activity before decreasing tropomyosin receptor kinase B activity

**FIGURE 1:**
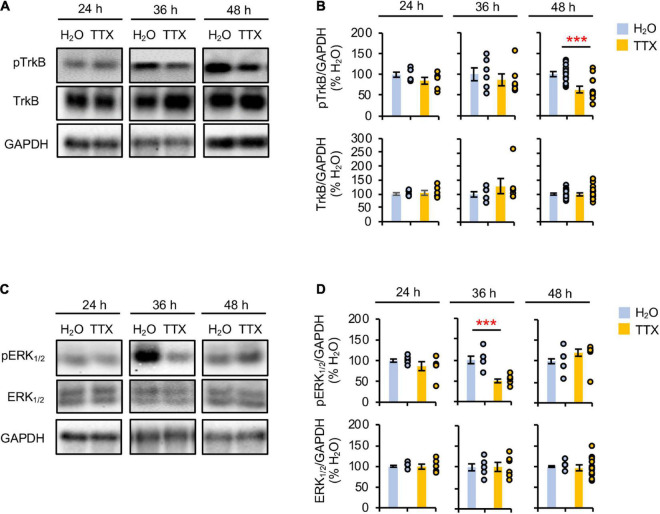
Prolonged activity blockade transiently reduces ERK1/2 activity before decreasing TrkB activity. Cultured hippocampal neurons (DIV 10) were treated for 24 h, 36 h, and 48 h with TTX (1 μM) to block their activity or its vehicle control (0.1% H_2_O), and subjected to immunoblot analysis for pTrkB (pTrkA^*Tyr*674/675/^pTrkB^*Tyr*706/707^) and TrkB **(A,B)** or pERK_1/2_ (pERK1^*Thr*202/Tyr204^/pERK2^*Thr*185/Tyr187^) and ERK_1/2_ **(C,D)**. GAPDH served as a loading control. **(A,B)** TTX treatment for 48 h decreased pTrkB level. **(A)** Representative immunoblots. **(B)** Quantification of pTrkB and total TrkB immunodensities. The immunodensity ratios (pTrkB/GAPDH and TrkB/GAPDH) were normalized to vehicle control. Number of culture dishes used: pTrkB (24 h: H_2_O = 6, TTX = 6; 36 h: H_2_O = 6, TTX = 6; 48 h: H_2_O = 13, TTX = 13), TrkB (24 h: H_2_O = 6, TTX = 6; 36 h: H_2_O = 6, TTX = 6; 48 h: H_2_O = 17, TTX = 20). **(C,D)** TTX treatment for 36 h decreased pERK_1/2_ level. **(C)** Representative immunoblots. **(D)** Quantification of pERK_1/2_ and total ERK_1/2_ immunodensities. The immunodensity ratios (pERK_1/2_/GAPDH and ERK_1/2_/GAPDH) were normalized to vehicle control. Number of culture dishes used: pERK_1/2_ (24 h: H_2_O = 6, TTX = 6; 36 h: H_2_O = 6, TTX = 6; 48 h: H_2_O = 12, TTX = 12), ERK_1/2_ (24 h: H_2_O = 6, TTX = 6; 36 h: H_2_O = 6, TTX = 6; 48 h: H_2_O = 12, TTX = 12). The Student’s *t*-test was used (***p < 0.005). Data shown represent the mean ± SEM with individual data points.

Prolonged inhibition of TrkB is shown to increase AP firing rate similar to prolonged activity blockade (Desai et al., 1999), implicating reduced BDNF-TrkB signaling in homeostatic scaling of intrinsic excitability. To test if prolonged activity blockade decreases TrkB activation, we treated rat hippocampal neuronal culture (DIV 10–12) for 24–48 h with either vehicle control (0.1% H_2_O) or TTX (1 μM) which blocks neuronal activity (Supplementary Figures 1A–D). 48 h TTX treatment was previously shown to induce homeostatic scaling of AP firing rate in cultured hippocampal neurons (Lee et al., 2015), although the same treatment did not induce homeostatic scaling of spontaneous network activity (Supplementary Figure 2). Immunoblot analysis was performed for phosphorylated TrkA and TrkB at Tyr^674/675^ and Tyr^706/707^ (pTrkA/B), respectively. Since TrkB and TrkC are the primary Trk receptors present in the hippocampus (Muragaki et al., 1995), the changes in pTrkA/B represent the phosphorylation of TrkB at Tyr^706/707^, which corresponds to TrkB activation upon BDNF binding (Huang and Reichardt, 2003). We found that 48 h, and not 24 and 36 h, TTX application reduced the pTrkA/B level compared to the control treatment by 62.7 ± 6.8% (*p* < 0.01, Figures 1A,B). Total TrkB expression was unchanged by 24–48 h TTX treatment (Figures 1A,B). These findings suggest that 48 h activity blockade decreases the TrkB activity.

Prolonged inhibition of NMDAR alone also leads to homeostatic scaling of intrinsic excitability and reduction in *BDNF* transcript (Lee et al., 2015). Since ERK1/2 acts downstream of NMDAR and regulates transcription of plasticity-related genes including *BDNF* (Adams and Sweatt, 2002; Thomas and Huganir, 2004), we next investigated whether prolonged activity blockade decreases the ERK1/2 activity by immunoblotting for the phosphorylation of ERK1 at Thr^202^/Tyr^204^ and ERK2 at Thr^185^/Tyr^187^ (pERK1/2) (Figures 1C,D), which leads to activation of ERK1/2 (Robbins et al., 1993; Zhou and Zhang, 2002). We found that TTX treatment for 36 h but not 24 and 48 h significantly decreased the pERK1/2 level by 49.6 ± 1.8% (*p* < 0.01) compared to vehicle treatment (Figures 1C,D). The total ERK1/2 level was unaffected by TTX treatment (Figures 1C,D). These findings suggest that prolonged activity blockade leads to a transient reduction in ERK1/2 activity before decreasing TrkB activity.

### Prolonged extracellular signal-regulated kinase 1/2 inhibition decreases mRNA and protein expression of K_v_7.3

**FIGURE 2:**
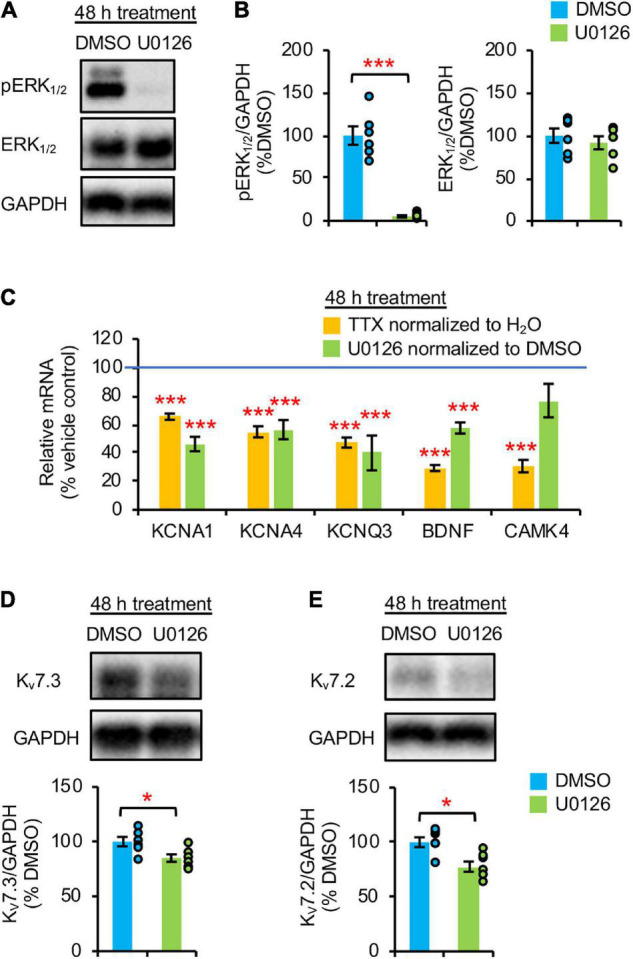
Prolonged pharmacological inhibition of ERK1/2 decreased mRNA and protein expression of K_v_7.3. **(A,B)** Cultured hippocampal neurons (DIV 10) were treated for 48 h with vehicle control (0.1% v/v DMSO) or 20 μM U0126. Immunoblot analysis was performed with antibodies for pERK_1/2_ (pERK1^*Thr*202/Tyr204^/pERK2^*Thr*185/Tyr187^), ERK_1/2_, and GAPDH (*n* = 6 per treatment). **(A)** Representative immunoblots. **(B**) Quantification of pERK_1/2_ and total ERK_1/2_ immunodensities normalized to vehicle control. **(C)** Both TTX and U0126 treatment for 48 h decreased *BDNF* and *KCNQ3* expression. Cultured hippocampal neurons (DIV 10) treated for 48 h with 1 μM TTX or its vehicle control (0.1% H_2_O), and 20 μM U0126 or its vehicle control (0.1% DMSO). QPCR was performed using the cDNA which was prepared from 2 μg of total RNA isolated from cultured neurons and validated primers for *Kcna1, Kcna4, Kcnq3, Bdnf, Camk4, and Gapdh* (*n* = 5 per treatment). Data were analyzed using the comparative threshold cycle (Ct) method and *Gapdh* internal control gene. Following normalization to *Gapdh* cDNA levels (which is reflected in the ΔCt values), the relative mRNA quantification (RQ) of the fold change for each condition compared to reference control was determined using the following equation: RQ = 2^(– Δ^ *^Ct)^*/2^(– Δ Ct reference)^. The RQ data is shown as mean ± SEM. One-way ANOVA with Tukey *post hoc* was used (***p < 0.005). **(D,E)** ERK1/2 inhibition for 48 h decreased K_v_7.3 and K_v_7.2 expression. Hippocampal cultured neurons (DIV 10) were treated for 48 h with 20 μM U0126 or its vehicle control (0.1% v/v DMSO) and subjected to immunoblot analyses with the verified antibodies for K_v_7.3 (Supplementary Figure 4) and antibodies for K_v_7.2 and GAPDH (*n* = 6 per treatment). **(D)** Representative Immunoblot and quantification of K_v_7.3. **(E)** Representative Immunoblot and quantification of K_v_7.2. The immunodensity ratios (K_v_7.3/GAPDH and K_v_7.2/GAPDH) were normalized to vehicle control. Data shown represent the mean ± SEM with individual data points. The Student’s *t*-test was used (*p < 0.05).

Homeostatic scaling of intrinsic excitability is associated with reductions in the gene expression of BDNF and multiple K^+^ channels including K_v_1 channels (*KCNA1, KCNA4*) and K_v_7 channels (*KCNQ3*) (Lee et al., 2015). Since this transcriptional down regulation and a decrease in TrkB activity occurs at 48 h TTX application after a transient reduction in ERK1/2 activity at 36 h (Figures 1C,D, 2C), we next tested if pharmacological inhibition of ERK1/2 for 48 h with a MEK1/2 specific inhibitor U0126 (20 μM) decreases *BDNF* and *KCNQ3* transcript levels. Since MEK1/2 phosphorylates and activates ERK1/2, MEK1/2 inhibition by U0126 leads to specific inhibition of ERK1/2 (Favata et al., 1998). Indeed, 48 h U0126 application induced a marked 95% reduction in pERK1/2 level without affecting total ERK1/2 expression compared to vehicle control (0.1% v/v DMSO), indicative of ERK1/2 inhibition (Figures 2A,B). Interestingly, U0126 application also decreased spontaneous activity, burst duration, and number of spikes per burst of cultured hippocampal neurons (Supplementary Figures 1E–G, 3F) without inducing homeostatic scaling of their spontaneous activity (Supplementary Figure 3).

Importantly, 48 h U0126 application decreased the transcript levels of *BDNF, KCNQ3, KCNA1*, and *KCNA4* by 42.6 ± 3.8%, 60.1 ± 12.2%, 53.9 ± 5.0%, and 43.8 ± 6.7%, respectively (*p* < 0.005) (Figure 2C) to a similar extent as 48 h TTX treatment (Figure 2C; Lee et al., 2015). In contrast, *CAMK4* expression was reduced only by 48 h application of TTX but not U0126 (*p* > 0.05) (Figure 2C), suggesting that ERK1/2 does not regulate the mRNA expression of CAMK4 implicated in homeostatic scaling of excitatory synaptic strength (Joseph and Turrigiano, 2017). Compared to DMSO, 48 h U0126 treatment also decreased K_v_7.3 and K_v_7.2 protein expression by 15.1 ± 4.1 and 22.5 ± 4.6%, respectively, (*p* < 0.05) (Figures 2D,E).

### Prolonged blockade of neuronal activity or extracellular signal-regulated kinase 1/2 decreases ankyrin-G and K_v_7.3 expression at the axon initial segment

**FIGURE 3:**
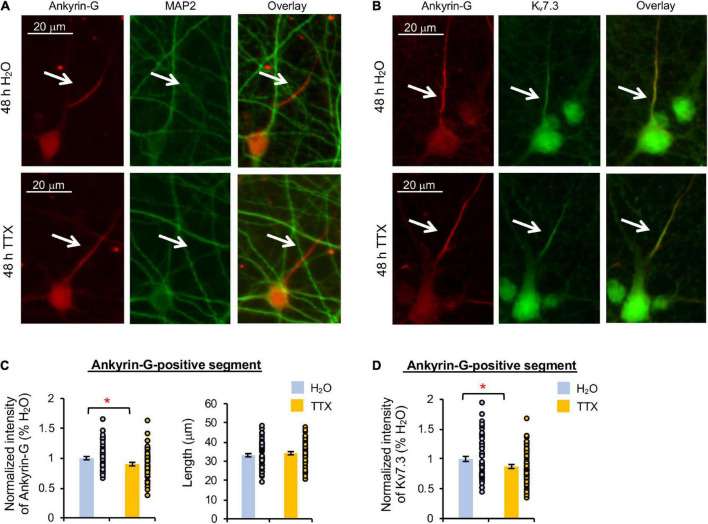
Prolonged activity blockade decreases K_v_7.3 and ankyrin-G expression at the AIS. Cultured hippocampal neurons (DIV 11–12) were treated for 48 h with 1 μM TTX or its vehicle control (0.1% H_2_O) and subjected to immunocytochemistry with antibodies for the AIS marker ankyrin-G, somatodendritic marker MAP2, or K_v_7.3. **(A)** Representative images of ankyrin-G and MAP2. **(B)** Representative images of ankyrin-G and K_v_7.3. **(C)** Quantification of the background-subtracted raw integrated fluorescent intensity of ankyrin-G-positive segment in the axon, which was normalized to vehicle control, and the length of the ankyrin-G-positive segment. **(D)** Quantification of the background-subtracted raw integrated fluorescent intensity of K_v_7.3 within the ankyrin-G-positive segment, which was normalized to vehicle control. Data shown represent the mean ± SEM with individual data points (H_2_O = 62 neurons from 46 images, TTX = 61 neurons from 43 images). Images were collected from 2 independent experiments. The Student’s *t*-test was used (*p < 0.05). Scale bar = 20 μm.

**FIGURE 4:**
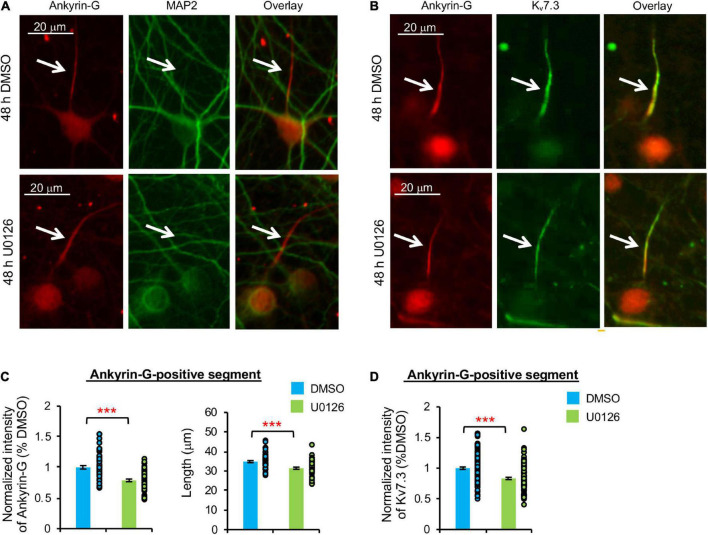
Prolonged pharmacological inhibition of ERK1/2 decreases K_v_7.3 and ankyrin-G expression at the AIS and the AIS length. Cultured hippocampal cultured neurons (DIV 11–12) were treated for 48 h with 20 μM U0126 or its vehicle control (0.1% DMSO) and subjected to immunocytochemistry with antibodies for the AIS marker ankyrin-G, somatodendritic marker MAP2, or K_v_7.3. **(A)** Representative images of ankyrin-G and MAP2. **(B)** Representative images of ankyrin-G and K_v_7.3. **(C)** Quantification of the background-subtracted raw integrated fluorescent intensity of ankyrin-G-positive segment in the axon, which was normalized to vehicle control, and the length of the ankyrin-G-positive segment. **(D)** Quantification of the background-subtracted raw integrated fluorescent intensity of K_v_7.3 within the ankyrin-G-positive segment, which was normalized to vehicle control. Data shown represent the mean ± SEM with individual data points (DMSO = 132 neurons from 69 images, U0126 = 126 neurons from 53 images). Images were collected from 2 independent experiments. The Student’s *t*-test was used (***p < 0.005). Scale bar = 20 μm.

Heteromeric K_v_7.2/K_v_7.3 channels are concentrated at the AIS by their interaction with ankyrin-G (Chung et al., 2006; Pan et al., 2006), which is an AIS-resident scaffolding protein critical for maintaining neuronal polarity (Pan et al., 2006; Leterrier, 2018). Therefore, we next examined if K_v_7.3 expression at the AIS is reduced by 48 h treatment with TTX or U0126. Immunostaining for ankyrin-G revealed the AIS, which does not overlap with the somatodendritic marker microtubule-associated protein 2 (MAP2) (Dehmelt and Halpain, 2005), demonstrating neuronal polarity and health regardless of the treatments (Figures 3A, 4A).

TTX application for 48 h induced a small but statistically significant reduction in K_v_7.3 immunodensity at the ankyrin-G-positive segment by 12.7 ± 3.5% compared to its vehicle control (*p* < 0.05) (Figures 3B,D). Ankyrin-G immunodensity was also reduced by 10.2 ± 2.9% (*p* < 0.05) in TTX-treated neurons without altering the length of the ankyrin-G-positive segment (Figures 3B,C). These findings indicate that prolonged activity blockade leads to a decrease in both K_v_7.3 and ankyrin-G expression at the AIS.

Similarly, 48 h U0126 treatment decreased ankyrin-G immunodensity by 21.2 ± 1.9% and the length of ankyrin-G-positive segment by 8.0 ± 5.5% compared to DMSO control (*p* < 0.005) (Figures 4A–C). K_v_7.3 immunodensity at the ankyrin-G-positive segment was also decreased by 16.8 ± 1.8% in U0126-treated neurons (*p* < 0.005) (Figure 4D), indicating that prolonged ERK1/2 inhibition leads to reductions in not only ankyrin-G and K_v_7.3 expression at the AIS, but also the AIS length.

## Discussion

Homeostatic scaling of intrinsic excitability is previously shown to involve reductions in *KCNQ3* transcript and K_v_7 current (Lee et al., 2015). However, the molecular mechanism underlying the homeostatic regulation of K_v_7 channels remains largely unknown. In this study, we provide evidence that prolonged activity blockade decreases ERK1/2 activity, followed by reductions in *BDNF* and *KCNQ3* transcripts, TrkB activation, and the expression of ankyrin-G and K_v_7.3 at the AIS (Figures 1, 2C, 3), the key site at which K_v_7 channels suppress AP firing in hippocampal neurons (Pan et al., 2006; Shah et al., 2008). Importantly, prolonged inhibition of ERK1/2 alone is sufficient to induce these reductions (Figures 2C–F, 4), revealing new mechanistic insights into homeostatic regulation of K_v_7 channels in response to prolonged activity blockade.

### Downregulation of extracellular signal-regulated kinase 1/2 activity as a potential signaling pathway for homeostatic control of K_v_7.3 and brain-derived neurotrophic factor-tropomyosin receptor kinase B signaling

ERK1/2 regulates gene transcription (Whitmarsh, 2007) by phosphorylating transcription factors (Hollenhorst, 2012) and links NMDAR activation with transcriptional modulation of key plasticity-associated proteins including BDNF (Medina and Viola, 2018). NMDAR stimulation induces ERK1/2 activation (Wu et al., 2001), which is required for the induction of long-term potentiation in the hippocampus (English and Sweatt, 1997; Rosenblum et al., 2002) and hippocampus-dependent learning and memory (Atkins et al., 1998; Blum et al., 1999). Furthermore, ERK1/2 facilitates protein synthesis critical for homeostatic scaling of excitatory synaptic strength (Rutherford et al., 1998; Wang et al., 2013). Despite the well-known roles of ERK1/2 in both Hebbian and homeostatic synaptic plasticity (Adams and Sweatt, 2002; Bateup et al., 2013), whether ERK1/2 contributes to homeostatic scaling of intrinsic excitability is unknown.

We have previously shown that 48 h TTX treatment leads to homeostatic scaling of AP firing rate in cultured rat hippocampal neurons (Lee and Chung, 2014; Lee et al., 2015). Here, we found that TTX application transiently decreased ERK1/2 activity by 36 h before reducing TrkB activation at 48 h (Figure 1). This is interesting since prolonged TrkB inhibition alone can induce homeostatic scaling of intrinsic excitability (Desai et al., 1999). Both TTX treatment and ERK1/2 inhibition by U0126 for 48 h also reduced *BDNF* and *KCNQ3* transcripts (Figure 2C), in line with a previous report that conditional deletion of MEK1/2, the kinase directly upstream of ERK1/2, decreases expression of ion channels and neurotransmitter receptors implicated in regulating neuronal excitability (Xing et al., 2016).

Interestingly, our MEA recording was unable to detect homeostatic scaling of spontaneous activity in response to TTX or U0126 treatment for 48 h (Supplementary Figures 2, 3), possibly due to the movement of the MEA dishes and manual removal of TTX-containing medium using pipettes. These physical processes can create uneven mechanical pressure that dislodges the neurons from the electrodes or alters their activity, contributing to variability that could have hindered the detection of homeostatic scaling of spontaneous activity. Nonetheless, since homeostatic scaling of AP firing rate can be induced by either prolonged TTX application or NMDAR inhibition (Lee and Chung, 2014; Lee et al., 2015) and ERK1/2 regulates transcription of *BDNF* downstream of NMDAR (Medina and Viola, 2018), our findings suggest that prolonged activity blockade may first decrease ERK1/2 activity due to reduced NMDAR activity, followed by downregulation of BDNF-TrkB signaling and K_v_7.3.

Prolonged ERK1/2 inhibition also decreased spontaneous activity, burst duration, and number of spikes per burst of cultured hippocampal neurons (Supplementary Figures 1D–F, 3F), consistent with the previous study reporting that 10–20 μM U0126 application decreases AP number by increasing AP half-width and decay time (Wang et al., 2018). The mechanism underlying the U0126-induced reduction in AP firing is unclear. U0126 may downregulate voltage-gated sodium current. Since U0126 is reported to inhibit the transient and sustained K^+^ currents such as *I*_*A*_ and *I*_*DR*_ (Wang et al., 2018), and such inhibition broadens AP half-width (Mitterdorfer and Bean, 2002), U0126 may reduce neuronal firing by regulating ERK1/2-dependent phosphorylation of K_v_4.2 or K_v_1.3 channels that contribute to *I*_*A*_ and *I*_*DR*_, respectively (Schrader et al., 2009; Jimenez-Perez et al., 2016). Although cautions must be exercised for using U0126 as a specific inhibitor for studying ERK1/2 signaling in neurons, a marked 95% reduction in the level of pERK1/2 upon 48 h U0126 treatment (Figures 2A,B) suggests that decreases in *BDNF* and *KCNQ3* transcripts are most likely induced by the inhibition of ERK1/2 activity (Figure 2C).

### Significance of homeostatic downregulation of K_v_7.3 at the axon initial segment

The computational modeling work has shown that activity-dependent regulation of ion channel transcripts can serve as one mechanism for neuronal homeostasis (O’leary et al., 2013). Indeed, homeostatic scaling of AP firing rate induced by prolonged activity blockade is associated with transcriptional downregulation of multiple K^+^ channels including axonally localized K_v_1 and K_v_7 channels (Lee et al., 2015). In this study, we found that activity blockade or ERK1/2 inhibition for 48 h decreased gene expression of *KCNA1*/K_v_1.1, *KCNA4*/K_v_1.4, and *KCNQ3*/K_v_7.3 (Figure 2C) and K_v_7.3 density at the ankyrin-G-positive segment (Figures 3, 4). Prolonged ERK1/2 inhibition also decreased the protein levels of both K_v_7.2 and K_v_7.3 (Figures 2D–E).

Heteromeric K_v_7.2/K_v_7.3 channels concentrate at the AIS (Pan et al., 2006) and this AIS localization is critical for their function to suppress AP firing in hippocampal neurons (Shah et al., 2008), whereas multiple epilepsy mutations decrease their enrichment at the AIS (Chung et al., 2006; Cavaretta et al., 2014; Kim et al., 2018; Zhang et al., 2020) and disrupt their ability to inhibit neuronal intrinsic excitability (Cavaretta et al., 2014; Kim et al., 2018). Heteromeric K_v_7.2/K_v_7.3 channels produce current amplitudes which are ∼10-fold larger (Wang et al., 1998; Hadley et al., 2000; Gamper et al., 2003; Maljevic et al., 2003; Schwake et al., 2003) and display significantly more surface expression at the AIS and axons of cultured hippocampal neurons than homomeric K_v_7.2 or K_v_7.3 channels do (Schwake et al., 2000; Chung et al., 2006). Thus, we propose that the reduction of K_v_7.3 density at the ankyrin-G-positive segment upon prolonged activity blockade or ERK1/2 inhibition (Figures 3, 4) is expected to decrease the surface density of K_v_7.2/K_v_7.3 channels and K_v_7.3 channels at the AIS, which in turn will reduce their current critical for suppressing AP firing.

### Mechanism underlying homeostatic downregulation of K_v_7.3 at the axon initial segment

How does prolonged activity blockade decrease K_v_7.3 density at the AIS? Reduction in K_v_7.3 mRNA and protein expression upon a decrease in ERK1/2 activity (Figures 1C,D, 2C,E) is one way to reduce K_v_7.3 density at the AIS. Since ankyrin-G binding to K_v_7.2 and K_v_7.3 mediates their enrichment at the AIS (Chung et al., 2006; Pan et al., 2006) and K_v_7.3 displays a stronger interaction with ankyrin-G than K_v_7.2 (Xu and Cooper, 2015), another possible way is to decrease ankyrin-G density at the AIS. Indeed, this is what we observed following both prolonged activity blockade and prolonged ERK1/2 inhibition (Figures 3A,C, 4A,C).

Interestingly, prolonged ERK1/2 inhibition also decreased the length of the ankyrin-G-positive segment, whereas prolonged activity blockade had no effect (Figures 4A,C). The mechanism underlying activity-dependent changes in ankyrin-G density and spread at the AIS is unclear. In cultured hippocampal neurons, BDNF activation is reported to regulate the AIS position (Guo et al., 2017), whereas ankyrin-G clustering at the AIS depends on its phosphorylation and subsequent recruitment of β4-Spectrin (Yang et al., 2019). Considering that chronic pharmacological inhibition of K_v_7 channels can also induce homeostatic regulation of AP firing rate (Lezmy et al., 2017) and the AIS is the site of activity-dependent remodeling (Yamada and Kuba, 2016), investigating the detailed mechanism underlying K_v_7.3 and ankyrin-G downregulation at the AIS during homeostatic scaling of intrinsic excitability warrants future studies.

## Data availability statement

The raw data supporting the conclusions of this article will be made available by the authors, without undue reservation. The data excel file and the immunoblot images are also available in figshare: doi: 10.6084/m9.figshare.20315379.v1 and doi: 10.6084/m9.figshare.20326608.v1.

## Ethics statement

The animal study was reviewed and approved by the Institutional Animal Care and Use Committee, University of Illinois at Urbana-Champaign.

## Author contributions

BB and HC conceived of the study, participated in its design and coordination, drafted the manuscript, and prepared figures. BB, HK, AW, EK, GT, and WO performed the experiments and data analysis. N-PT trained BB in MEA recording and analysis and provided MEA equipment. All authors contributed to the article and approved the submitted version.

## Conflict of interest

The authors declare that the research was conducted in the absence of any commercial or financial relationships that could be construed as a potential conflict of interest.

## Publisher’s note

All claims expressed in this article are solely those of the authors and do not necessarily represent those of their affiliated organizations, or those of the publisher, the editors and the reviewers. Any product that may be evaluated in this article, or claim that may be made by its manufacturer, is not guaranteed or endorsed by the publisher.
