# Supplementary material for: Homeostatic regulation of extracellular signal-regulated kinase 1/2 activity and axonal Kv7.3 expression by prolonged blockade of hippocampal neuronal activity
Source: Front Cell Neurosci. 2022 Jul 28;16:838419. doi: 10.3389/fncel.2022.838419 (PMC9366003; doi:10.3389/fncel.2022.838419)
Supplement: Supplementary file 1 [file Data_Sheet_1.pdf]

Supplementary Material

Homeostatic regulation of ERK1/2 activity and axonal Kv7.3 expression by prolonged blockade of hippocampal neuronal activity

Brian C. Baculis[1,2], Harish Kesavan[2], Amanda C. Weiss[1,2], Edward H. Kim[2], Gregory C. Tracy[2], Wenhao Ouyang[2], Nien-Pei Tsai[1,2], and Hee Jung Chung[1,2,3,4*]

[1]Neuroscience Program, University of Illinois at Urbana-Champaign, Urbana, IL, USA

[2]Dept. of Molecular and Integrative Physiology, University of Illinois at Urbana-Champaign, Urbana, IL, USA

[3]Beckman Institute for Advanced Science and Technology, University of Illinois at Urbana-Champaign, Urbana, IL, USA

[4]Institute of Genomic Biology, University of Illinois at Urbana-Champaign, Urbana, IL, USA

*** Correspondence:**

Hee Jung Chung

Department of Molecular and Integrative Physiology,

University of Illinois at Urbana-Champaign,

407 South Goodwin Avenue, 524 Burrill Hall,

Urbana, IL 61801, USA.

chunghj@illinois.edu

1 Supplementary Figures and Tables

1.1 Supplementary Figures

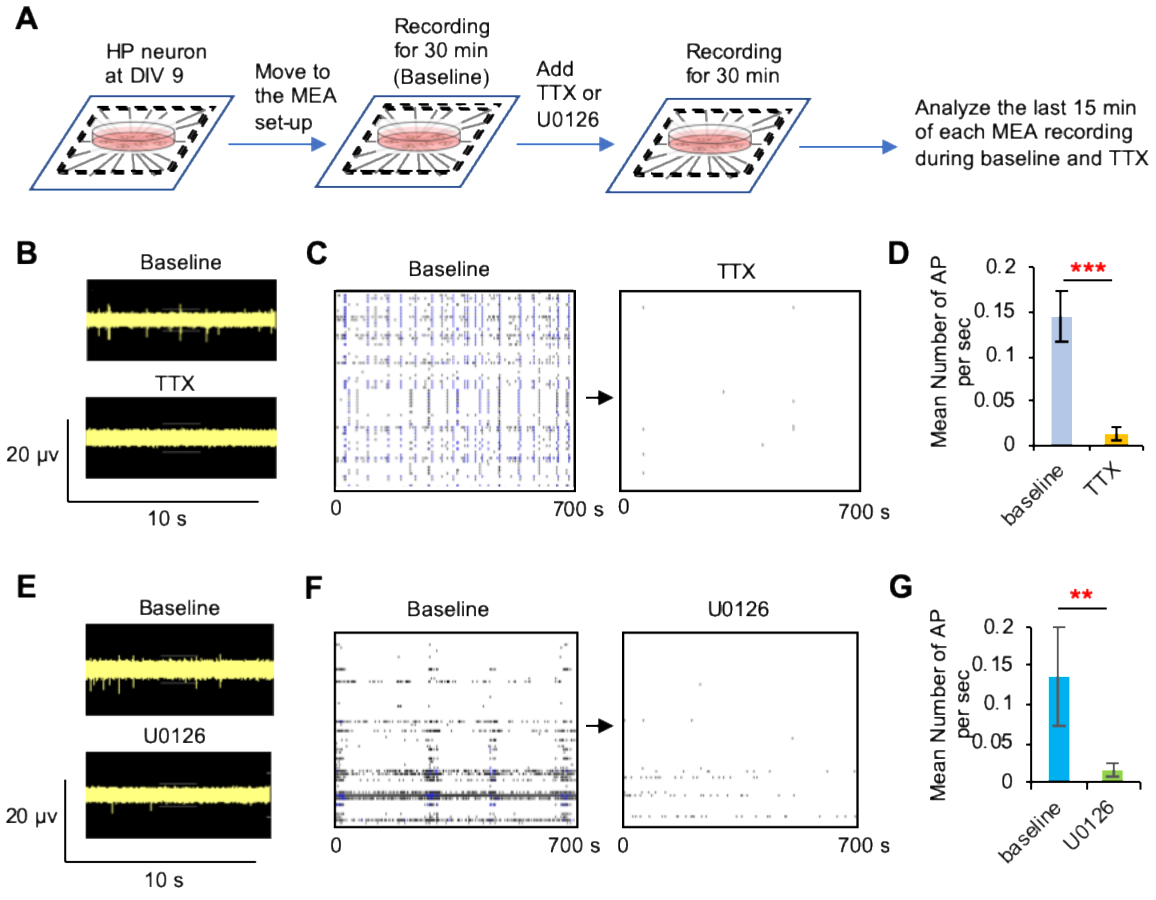

Supplementary Figure 1. MEA recording of spontaneous activity of cultured hippocampal neurons following acute TTX or U0126 treatment. (A) Schematic of MEA recording of cultured hippocampal neurons (DIV 9) in the Axion Muse system before (baseline) and immediately after acute application of TTX (1 µM) or U0126 (20 µM). Recordings consisted of 30 min. **(B-G)** MEA recordings of spontaneous neuronal activity following acute drug treatment. The AxIS software was used to extract action potentials (APs) which were differentiated from noise using adaptive threshold crossing method. Since treatment of medium and physical movement of the MEA plates is shown to perturb network activity of neurons on the plate, the first 15 min of recording data were omitted from the analyses. Because of the variability of the basal neuronal activity from one dish to another, the activity after treatment was normalized to baseline (before treatment) for each dish. **(B-D)** Confirmation of activity blockade by TTX treatment. **(B)** Sample traces of spikes. **(C)** Rosta plot of spikes. **(D)** Quantification of the mean number of APs per second. **(E-G)** Acute application of U0126 decreased spontaneous activity of cultured hippocampal neurons. **(E)** Sample traces of spikes. **(F)** Rosta plot of spikes. **(G)** Quantification of the mean number of APs per second. **(D, G)** Data shown represents the mean ± SEM from 64 electrodes from 1 MEA plate. The Student's t-test was used (***$p < 0.005$, **$p < 0.01$).

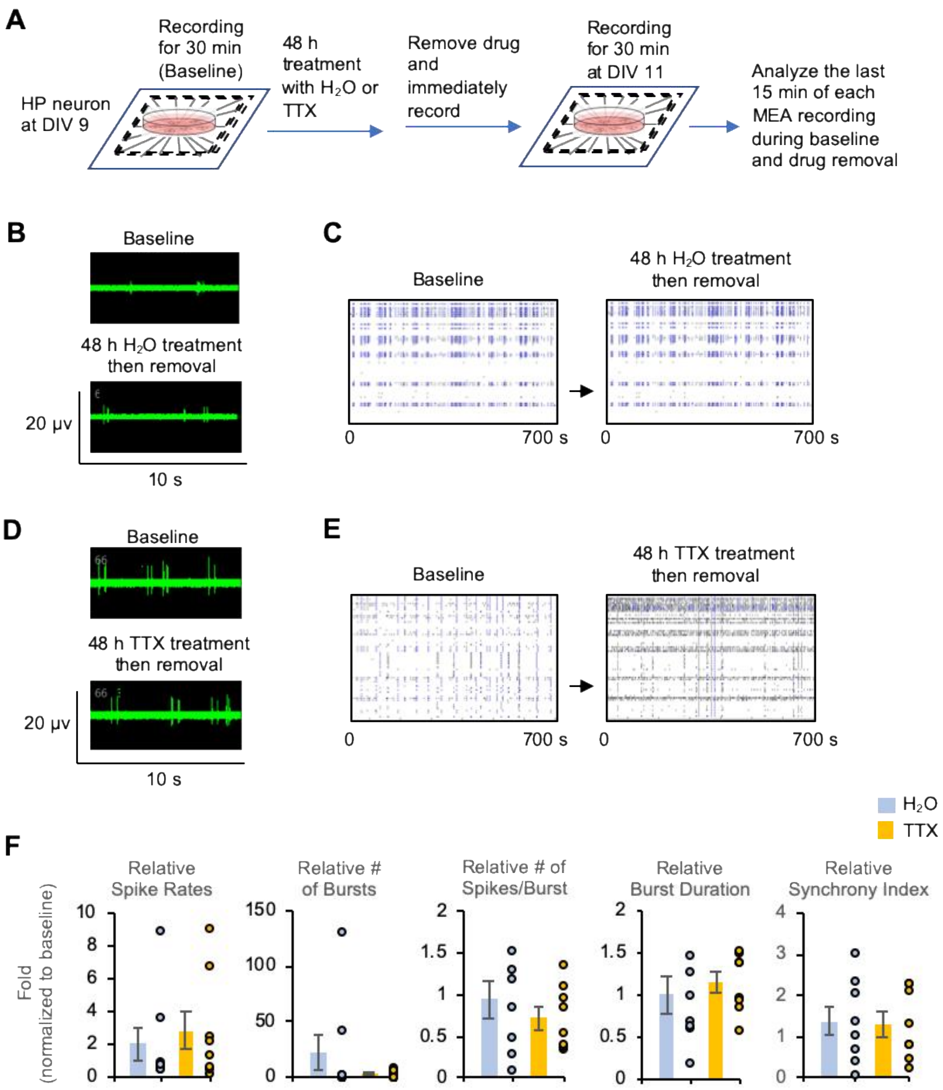

Supplementary Figure 2. MEA recording to observe homeostatic change in spontaneous activity of cultured hippocampal neurons following the withdrawal of TTX after its pre-treatment for 48 h. (A) Schematic of MEA recording of cultured hippocampal neurons in the Axion Muse system at DIV 9 before (baseline) and after 48 h treatment with TTX (1 µM) or its vehicle control (0.1% v/v H_2O) and immediately after the withdrawal of TTX. **(B-F)** 48 h TTX treatment did not induce homeostatic change in spontaneous activity of culture hippocampal neurons. The AxIS software was used to extract action potentials (APs). **(B, D)** Sample traces of spikes. **(C, E)** Rosta plot of spikes. **(F)** Data analyses of MEA recordings of the last 15 min of recording. Adaptive threshold crossing method was used for single spike detection settings to determine spike activity from noise. Inter-spike interval (ISI) threshold algorithm was used for burst detection settings with maximum ISI of 100 ms and minimum 5 spikes per single burst. Since treatment and removal of medium as well as physical movement of the MEA plates before and after recording are shown to perturb neuronal activity on the plate, the first 15 min of recording data were omitted from the statistical analyses. Because of the variability of the basal neuronal activity from one dish to another, the activity after treatment was normalized to baseline (before treatment) for each dish to indicate "Fold" change. Data shown represents the mean ± SEM. The number of MEA dishes used: n = 8 for TTX, n = 8 for H_2O. The Student's t-test was used.

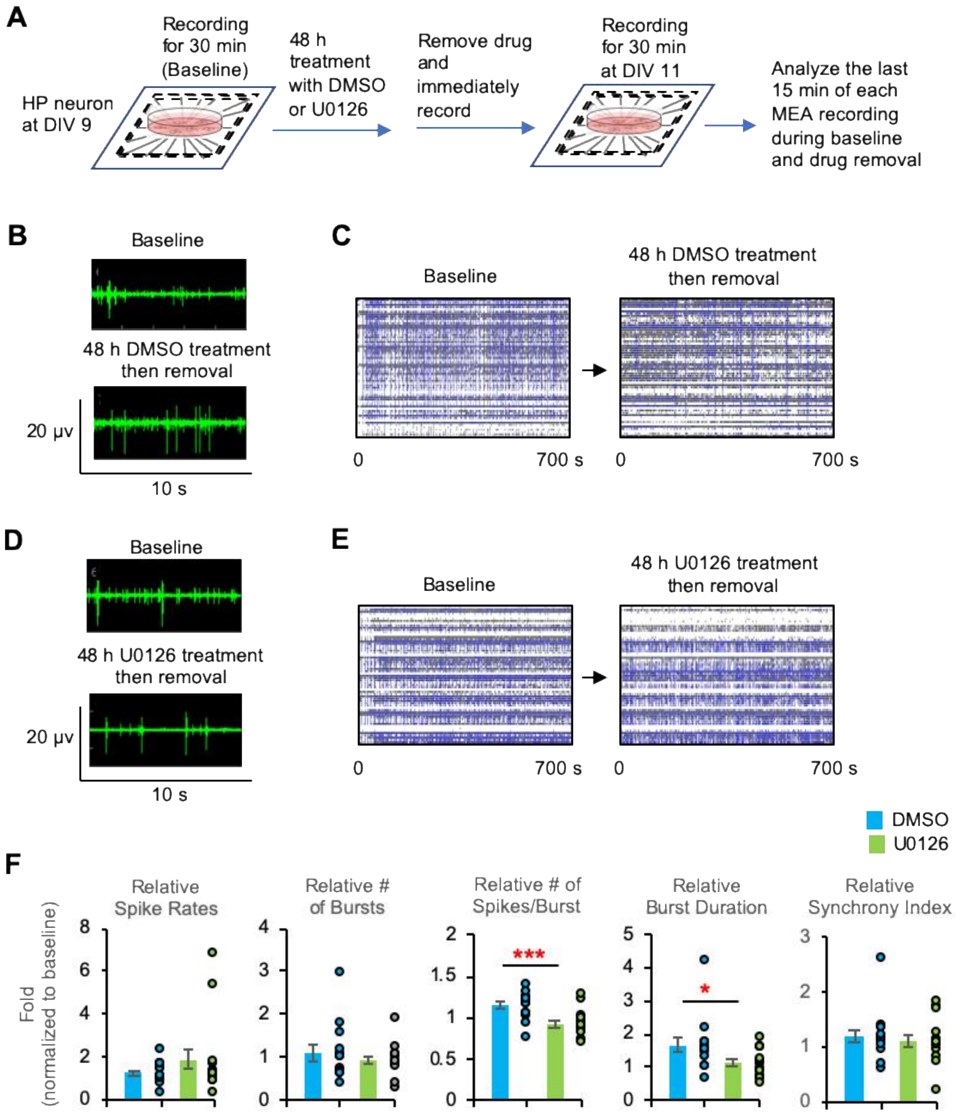

Supplementary Figure 3. MEA recording to observe homeostatic change in spontaneous activity of cultured hippocampal neurons following the withdrawal of U0126 after its pre-treatment for 48 h. (A) Schematic of MEA recording of cultured hippocampal neurons in the Axion Muse system at DIV 9 before 48 h treatment with U0126 (20 µM). or its vehicle control (0.1% v/v DMSO) (baseline), at DIV 11 after 48 h treatment, and immediately after the withdrawal of U0126. Recordings consisted of 30 min. **(B-F)** 48 h U0126 treatment induced homeostatic decrease in the number of spikes per burst and burst duration in culture hippocampal neurons. The AxIS software was used to extract action potentials (APs. Since treatment and removal of medium as well as physical movement of the MEA plates before and after recording are shown to perturb network activity of neurons on the plate, the first 15 min of recording data were omitted from the statistical analyses. (**B, D)** Sample traces of spikes. (**C, E)** Rosta plot of spikes. (**F**) Data analyses of MEA recordings of the last 15 min of recording. Because of the variability of the basal neuronal activity from one dish to another, the activity after treatment was normalized to baseline (before treatment) for each dish. Data shown represents the mean ± SEM. The number of MEA dishes used: n = 12 for U0126, n = 12 for DMSO. The Student's t-test was used (***$p < 0.005$, *$p < 0.05$).

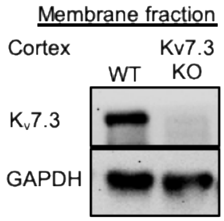

Supplementary Figure 4. Immunoblot analysis of cortices of K_v7.3 knock-out mice and wild-type mice to verify the specificity of anti-K_v7.3 antibody. Cortical tissues of K_v7.3 knock-out and wild type mice were fractionated into membrane (P2) and soluble (S2) lysate fractions, and the membrane fractions were subjected to immunoblotting with anti-K_v7.3 and anti-GAPDH antibodies.

1.2 Supplementary Methods

Multi-electrode array (MEA) recordings

Primary dissociated rat hippocampal neurons (1.5 x10^5 cells per plate) were plated onto poly D-lysine (0.1 mg/mL, Sigma)-coated MEA electrode area containing 64 electrodes in a MEA dish (Axion Biosystem, Catalog #M64-GL1-30Pt200 or Axion Biosystem, Catalog #M384-tMEA-6W-5). These neurons were maintained as described above. All drug treatments and MEA recordings were performed in media. To examine the effects of acute TTX and U0126 treatment on spontaneous network activity, neurons (DIV 9) in the MEA plates were placed in the MEA recording incubator (5% CO_2, 37°C), and field potentials at each MEA electrode relative to a ground electrode were recorded for 30 minutes (min) with a sampling rate of 25 kHz using Axion Muse 64 channel system (Axion Biosystem's Maestro Edge). It should be noted that MEA recordings presented in Supplementary Figures 1-2 were performed on previous Axion Biosystem MEA model from U0126 apart from acute treatment. Following this "baseline" recording, TTX or its vehicle control (H_2O) was added to the original medium to the final concentration of 1 μM or 0.1% v/v, respectively, and then field potentials were immediately measured for another 30 min recording (Supplementary Figure 1A). The same recording was repeated for 20 μM U0126 and its vehicle control (0.1% v/v DMSO). MEA recordings presented in Supplementary Figure 1 were performed on a previous model of Axion Muse 64 channel system (Axion Biosystem).

To examine homeostatic changes in neuronal network activity induced by 48 h TTX and U0126 application, the baseline recording was first performed at DIV 9 for 30 min. The neurons were put back in the cell culture incubator for 48 h drug application with drugs refreshed every 24 h. For MEA recording after drug withdrawal at DIV 11, the media containing TTX, U0126, or their respective vehicle control was replaced with the "saved" medium from the last feeding before the drug treatment

and field potentials were recorded for 30 min. MEA recordings of TTX-treated neurons presented in Supplementary Figure 2 were performed on previous Axion Biosystem MEA model from U0126 apart from acute treatment, whereas MEA recordings of U0126-treated neurons presented in Supplementary Figure 3 were performed on the current (new) Axion Biosystem Muse 64 channel system.

AxIS software (Axion Biosystems) was used for the extraction of spikes (i.e. action potentials) from the field potentials obtained from the Axion Muse system. A spike was defined as the activity exceeding a threshold of ± 6 standard deviations which was independently set for each channel after filtering. Because changes in network activity can be caused by physical movement of the MEA plates (i.e. taking the plates out of the cell culture incubator and placing them in the MEA recording chamber), only the last 15 min of each recording was used in data analyses. The setting for burst detection was a minimum of 5 spikes with a maximum inter-spike interval of 0.1 s. Synchrony index was computed through AxIS software by taking the cross-correlation between two spike trains, removing the portions of the cross-correlogram that are contributed by the auto-correlations of each spike train, and reducing the distribution to a single metric. A value of 0 corresponds to no synchrony and a value of 1 corresponds to perfect synchrony. Raster plots were generated using NeuralMetricTool software.

Biochemical fractionation of mouse cortices

$KCNQ3^{-/-}$ knock-out mice and wild-type littermates have been previously described (Tzingounis and Nicoll, 2008) and their fresh frozen brain tissues were kind gifts from Dr. Anastassios V. Tzingounis (University of Connecticut). The brain tissues of a $KCNQ3^{-/-}$ knock-out mouse and a wild-type mouse were collected at 1.5 months of age, and fast frozen. Cortices were dissected and were biochemically fractionated in ice-cold homogenization buffer (solution A) containing (in mM): 320 sucrose, 1 NaHCO3, 1 MgCl2, 0.5 CaCl2, 0.4 HEPES (pH 7.4) and Halt protease inhibitors (Thermo Fisher Scientific). The resulting cell lysate was subject to centrifugation at 800g for 10 min at 4°C to separate

soluble (S1) supernatant from insoluble (P1) pellet. S1 was then centrifuged at 13,800g for 10 min at 4°C to separate soluble proteins (S2) from the pellet (P2) which contains membrane-bound proteins. The P2 fraction was then resuspended in ice-cold (solution B) containing (in mM): 160 sucrose, 6 Tris-HCl, 0.5% Triton-X (pH 8.0) and Halt protease inhibitors. BCA assay (Pierce) was performed to determine protein concentrations of the S2 and P2 fractions, which were subsequently normalized to 0.5 mg/mL in Solutions A and B, respectively, and stored at −80°C until use for immunoblot analysis.

Immunoblot Analysis

Samples (hippocampal neuronal lysates or the P2 fractions of mouse cortices) were mixed with 5X SDS sample buffer in 1:5 ratio and heated at 75°C for 30 min. The SDS sample buffer contained (mM): 75 Tris, 50 TCEP, 0.5 EDTA, 10% SDS, 12.5% glycerol, 0.5 mg/mL Bromophenol Blue. The samples were then run on 4%-20% gradient SDS-PAGE gels (Bio-Rad) and transferred to a methanol-treated polyvinylidene difluoride (PVDF) membrane (Millipore). Immunoblot analysis was performed on the PVDF membrane. In brief, the membrane was blocked for 1 h in blocking buffer (1% milk and 0.1% Tween-20 in TBS) and incubated overnight at 4°C with primary antibodies for $K_v7.3$ (1:500), ERK1/2-$pTyr^{202/204}$, ERK1/2, TrkA-$pTyr^{674/675}$/TrkB-$pTyr^{706/707}$, TrkB, and GAPDH (all 1:1000). GAPDH was used as a loading control. After the membranes were incubated with horseradish peroxidase-conjugated secondary antibodies (1:200) in blocking buffer for 1 h and washed, they were exposed to enhanced chemifluorescence substrate (ECL, Thermo Fisher Scientific). Luminescent signals were detected using an autoradiography film on a Konica SRX-101A film processor or Life Technologies iBright CL1000 (Thermo Fisher Scientific) imaging system and quantified by ImageJ software (National Institutes of Health). To quantify the immunoblots, the mean grey values of immunoblot bands were measured from the inverted images using a rectangle region of interest (ROI) tool in ImageJ software to obtain positive pixel values. The mean grey value from the ROI of the same size was also obtained

from the immunoblot area that contained no noticeable bands as “background”. The background-subtracted immunoblot band intensity of the protein of interest was first divided by that of GAPDH control protein. The ratio (protein of interest/GAPDH) of the vehicle control was used as 100% and the drug treatment (TTX or U0216) were normalized to the vehicle control.
